# Bariatric surgery and mental health outcomes: an umbrella review

**DOI:** 10.3389/fendo.2023.1283621

**Published:** 2023-11-02

**Authors:** Saikam Law, Shiliang Dong, Fuqing Zhou, Dexi Zheng, Cunchuan Wang, Zhiyong Dong

**Affiliations:** ^1^ Department of Metabolic and Bariatric Surgery, First Affiliated Hospital of Jinan University, Guangzhou, China; ^2^ School of Medicine, Jinan University, Guangzhou, China; ^3^ Gernaral Surgery, Dancheng County People’s Hospital, Zhoukou, China

**Keywords:** mental health, bariatric surgery, obesity, umbrella review, meta-analysis

## Abstract

**Aims:**

To evaluate the breadth, depth and effectiveness of the evidence quality of all existing studies on bariatric surgery and mental health outcomes.

**Design:**

Umbrella review of existing Systematic review and meta-analyses.

**Data sources:**

PubMed, Embase, Web of Science, and the Cochrane Liberally databases of Systematic review and meta-analyses, and hand searching the reference lists of eligible publications.

**Results:**

The search identified nine studies and 20 mental health outcomes from 1251 studies. Evidence shows that bariatric surgery is associated with significant improvement in areas such as anxiety, depression and eating disorders (including binge-eating disorder), and there is a significant harmful association with suicide, self-harm and alcohol use disorder (AUD). Among them, the most studied outcome is depression (4 articles). High-quality evidence proves that the score of depressive symptoms can be significantly improved after bariatric surgery within a two-year follow-up period and is not affected by the follow-up time. Low-quality evidence shows that bariatric surgery can significantly reduce depressive symptoms regardless of age and BMI, with an odds ratio (OR) of 0.49. Regardless of the postoperative BMI, the anxiety symptoms of women over 40 still decreased significantly, with an OR of 0.58. Regardless of the type of surgery, surgery can significantly reduce the incidence of eating disorders and symptoms. However, there is no obvious change in the follow-up time of AUD in the first two years after bariatric surgery, and the risk increases obviously in the third year, with an OR of 1.825. The evidence of moderate research shows that the risk of suicide and self-harm increases after bariatric surgery. The odds ratios in the same population and the control group were 1.9 and 3.8 times, respectively.

**Conclusion:**

Bariatric surgery is beneficial for improving most mental health-related outcomes. However, we should be cautious about the increased risk of adverse mental health after surgery, such as suicide, self-harm, and AUD.

## Introduction

Obesity has become one of the most severe global public health problems of the 21^st^ century—more than 35% of men and 40% of women with obesity in the United States (1, 2). Obesity may be associated with social stigma and self-potential shame, causing a psychological burden on patients with obesity and may increase with body mass index (BMI) (3). There has been growing evidence of a two-way association between mental illness and obesity, particularly among bariatric surgery candidates (4). Depression and anxiety are common mood disorders that often coexist with obesity (5). In the general United States population, the prevalence of depression in patients with obesity seeking surgery exceeds the published prevalence (19% VS 8%) (5). Depression has long been thought to be significantly associated with binge-eating disorders (BED) (6). Eating disorder is associated with an additional burden on patients with obesity, Such as low quality of life, impulsivity, and emotional regulation disorders (7), in the regulation of hunger/satiety (8), on the palate (9), on food preferences or intolerances (10, 11), and a higher likelihood of coexisting mental health conditions.

Although bariatric surgery has been shown to alleviate complications related to physical health, its effects on mental illness have not been clearly articulated (12). In the United States, the rate of bariatric surgery remains < 1% among the eligible population (13). This low rate may be due to questions about the long-term effectiveness of bariatric surgery (14). Bariatric surgery was associated with significant improvements in depression, BED (15, 16), and eating disorders (17). While the results were encouraging, with long-term follow-up data suggesting that some postoperative patients did not experience psychological benefits or reported increased rates of depression and BED recurrence (16, 18). People are concerned about the potential risks of mental health disorders after bariatric surgery, including self-harm, suicide, and substance abuse (19, 20). One study reported that patients after bariatric surgery had a 1.98-fold increased risk of suicide compared to usual care of patients with obesity (21). The incidence of suicide and suicide attempts occurs on average 3.8 to 3.9 years after surgery (22). In addition, the incidence of self-harm and alcohol use disorders (AUD) increases postoperatively (23, 24) and may be associated with changes in reward mechanisms after bariatric surgery (25). we re-evaluate the meta-analysis/systematic review of all the mental health outcomes of bariatric surgery to reveal the quality and strength of the evidence.

## Method

### Search strategy

**Figure 1:**
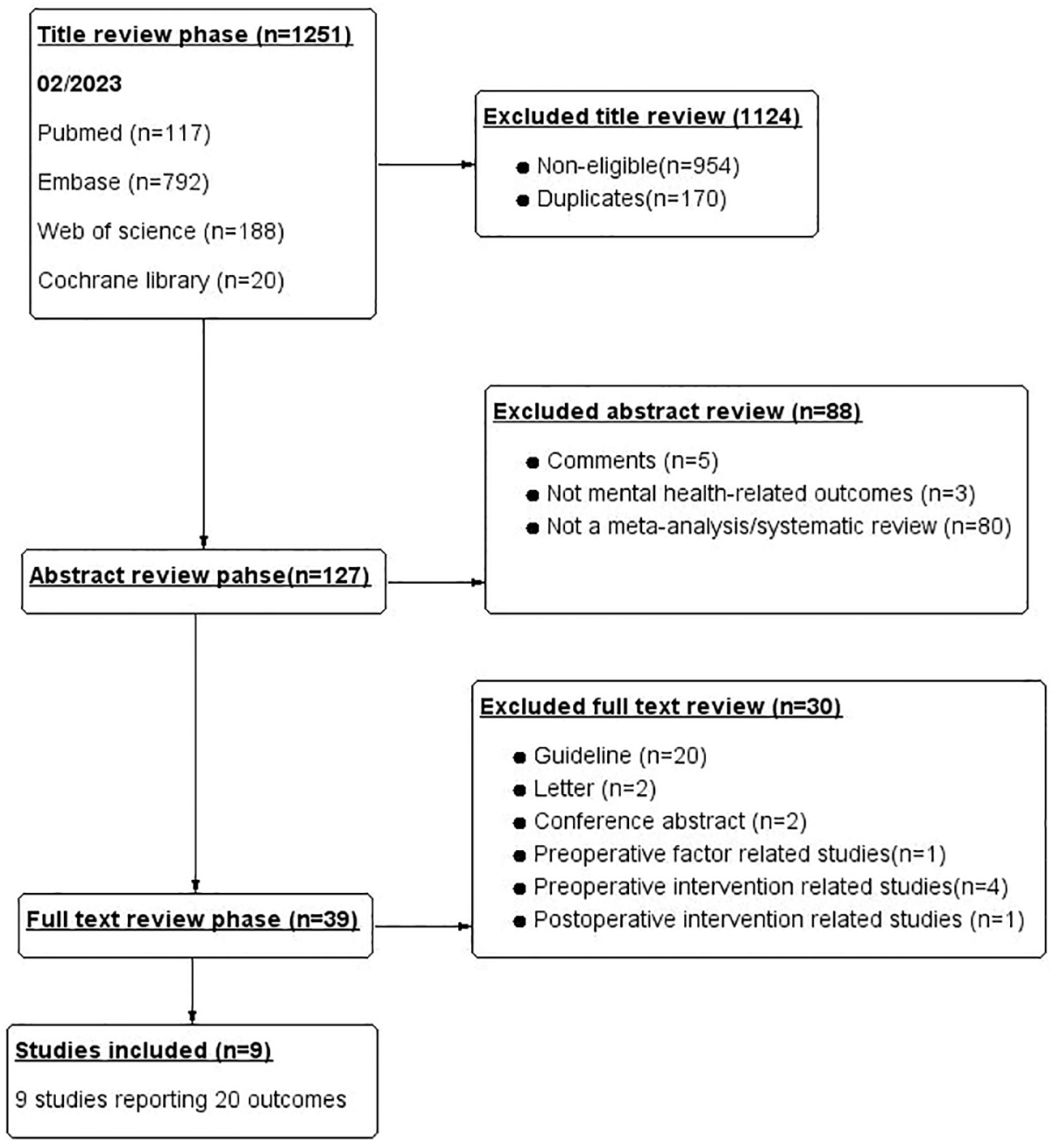
Flowchart of the article selection process.

This umbrella evaluation is completed according to the previous standardised procedures (26, 27). SL, SD, and FZ systematically searched PubMed, Embase, Web of Science, and the Cochrane Library databases until February 1, 2023. Within each theme “or” operators were used to combine terms, the “and” operator is used between different topics. Searches were tailored according to the functionality of each database. Where possible, MeSH terms were used that corresponded to the thematic areas. To avoid missing relevant meta-analyses during the initial search, we hand-searched the reference lists of eligible publications. The flowchart of the selection process is shown in **Figure 1**. We used ‘bariatric surgery’ and ‘mental health’ and ‘meta-analysis/systematic review’ and their synonym as keywords in the search database. Because mental health is disturbed by a wide range of external and internal factors, we choose the most relevant and common mental illness related to bariatric surgery as the classification of the psychological outcome, including anxiety, depression, suicidality, suicidal ideation, posttraumatic stress disorder (PTSD), substance abuse disorders, personality disorders, and eating disorders—primarily BED. No restrictions were placed on language. The detailed search strategy is in **Supplementary File S1**.

### Eligibility criteria

The inclusion and exclusion criteria for this umbrella review are as follows: The literature included in this review is selected as follows:(1) The study reported the results of the relationship between bariatric surgery and mental health; (2) Each outcome includes at least three studies; (3) Study on the effective quantity of research report: Odds ratio (OR), Relative risk (RR), standardised mean difference (SMD), prevalence rate (PR), and Hazard Ratio (HR); (4) Aggregate effect quantity is 95% confidence interval (CI); (5) Conducted a systematic review with meta-analysis. Without adding any language restrictions, we excluded studies that could not extract data, such as guidelines, review articles, animal studies, case reports, letters, posters, conference abstracts, systematic review protocols, and other basic studies and book chapters.

### Study selection

SL and FZ independently screened titles and abstracts, which decided on the full text of systematic reviews/meta-analyses that may meet the requirements. Disagreements are discussed by the third author (SD) and resolved by consensus. For any article that needs to be translated into English, online translation software (Google Translation) is used at first because it shows reasonable accuracy (28). SL recorded the research choice of systematic review according to the PRISMA statement based on systematic review and meta-analysis (29). Patient/population, intervention, comparison, outcome, and study design (PICOS) criteria are as follows (**Table 1**).

**Table 1:** PICOS criteria used to define the research question.

PICOS	Description
Participants	Population with obesity impacted by the bariatric surgery
Interventions	Bariatric surgery
Comparisons	Pre-post-surgery/non-surgery
Outcomes	Impacts mental health outcomes after bariatric surgery, for example, depression, anxiety posttraumatic stress disorder PTSD, personality disorders, substance abuse disorders suicidality or suicidal ideation, and eating disorders—primarily binge eating disorder
Study Design	Conducted as a systematic review with meta-analysis and included mental health outcomes

### Data extraction

SL, SD, and FZ extracted all data independently. Differences are resolved through discussion and consensus. When meta-analyses included multiple outcomes, each outcome was extracted separately. Extract the following data from the final included article: Mental health-related outcomes, first author and publication year, follow-up, type of bariatric surgery, types of meta-analysis and subjects’ number, the original study design, the metric of effect size, the effective model of meta-analysis, the effect size of 95%CI, the Heterogeneous P-value or I^2^, and publication bias.

### Assessment of methodological quality

SL, SD, and FZ independently used AMSTAR-2 to evaluate the methodological quality of each meta-analysis. The AMSTAR-2 tool provides a comprehensive critical evaluation tool to evaluate the systematic review of health interventions (30). AMSTAR-2 consists of 16 items, 7 of which are key areas. Each review was scored on whether there were methodological flaws in key or non-critical items. The grades are ‘High’, ‘Moderate’, ‘Low’ and ‘Critically low’. Disagreements were resolved through discussions, although a provision had been made to consult a fourth reviewer (ZD) if necessary.

### The credibility of the evidence

SL, SD, and FZ independently evaluated the quality of mental health-related outcomes using the GRADE framework (31). According to the assessment of inconsistency, risk of bias, indirectness, inaccuracy, and publication bias of each outcome, the quality of evidence is divided into four categories (‘high’, ‘medium’, ‘low’, and ‘very low’) (32).

### Data analysis

The purpose of the Umbrella Review was not to perform a meta-analysis of included studies, such as assessing study eligibility or the risk of bias, but rather to use relevant details extracted from the included meta-analysis to generalise the results for specific questions. It provides the existing and highest-level evidence about medical research and draws more reliable conclusions (26). We extracted only the existing effect size and 95% confidence interval for each outcome, which provided a manageable range of effect sizes for the study. Heterogeneity is expressed in P-values or I^2^ values, with P-values <0.1 or I^2^ ≥50% considered significant heterogeneity. Publication bias P-values< 0.1 were statistically significant.

### Corrected covered area index

When several meta-analyses investigated the same outcome, we conducted a corrected covered area index analysis on the repeated included literature. The degree of overlap in studies was assessed and calculated via the corrected covered area (CCA) index method (33). CCA within the range 0%–5% express a slight overlap, 6%–10% express a moderate overlap, 11%–15% express a high overlap, and >15% express a very high overlap (34) (**Table 2**).

**Table 2:** The overlapping included systematic reviews and meta-analyses.

Outcomes	Number of reviews	Number of included studies	CCA statistic (%)	Degree of overlapping
Depress symptoms	4	115	5.78%	slight
Suicide	2	67	1.52%	slight

## Result

### Literature review

**Figure 2:**
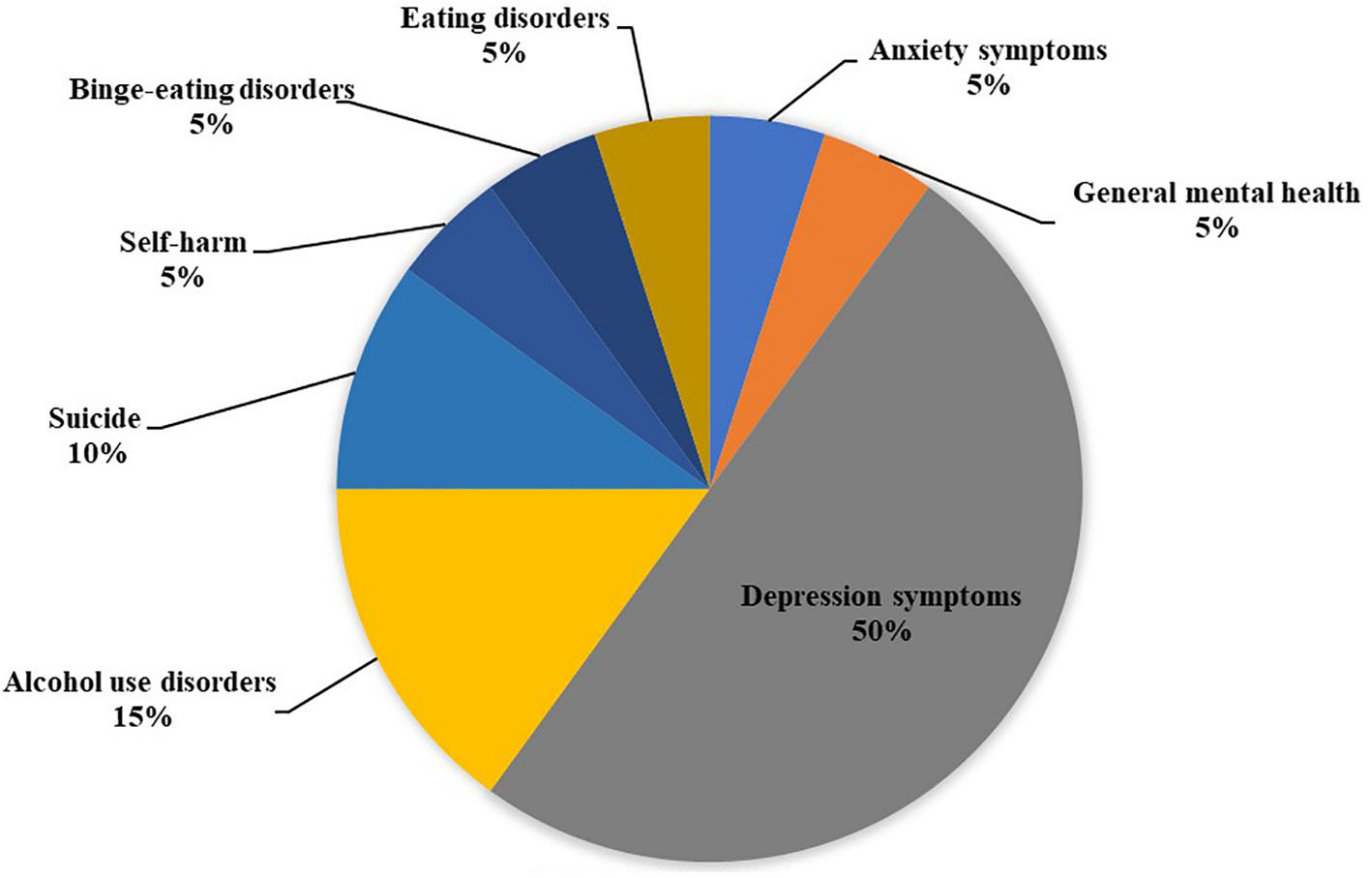
Map of mental health outcomes associated with bariatric surgery.

A total of 1251 potentially eligible articles were identified: 117 from PubMed, 188 from Web of Science, 792 from Embase, and 20 from Cochrane Library. We screened the titles of 1251 potential studies, excluding 170 duplicate articles and 954 studies not related to studies. We read abstracts, assessed the eligibility of the remaining 127 studies, excluded 88 studies, and classified the studies according to reasons for exclusion. Finally, nine eligible studies were selected by reading the full text, and 30 other studies that did not meet the requirements were excluded. The flow chart of the selection process is shown in **Figure 1**. Finally, nine studies with 20 different mental health-related outcomes were included in the umbrella review. **Figure 2** lists the main mental health outcomes after bariatric surgery. The relationship between bariatric surgery and mental health-related outcomes is shown in **Table 3**.

**Table 3:** Relationship between bariatric surgery and mental health-related results.

Outcome	First author; Year	Comparison	Type of bariatric surgery	Follow-up	Study type	No. of studies; Partiacipnts	Metric of MA	Effects model	Effect size	95% CI	I^2^%	Publication bias
General mental health	Gadd, N.2020	Pre-post surgery	IGB	6-76 months	cohort/RCT	5 (367)	SMD	REM	0.86	0.29 to 1.42	92%	NR
Alcohol use disorder	Azam, 2018	Pre-post surgery	Mix	3 years	cohort/case-control	8 (NR)	OR	REM	1.83	1.53 to 2.178	NR	NR
Alcohol use disorder	Azam, 2018	Pre-post surgery	Mix	1 year	cohort/case-control	8 (NR)	OR	REM	1.004	0.92 to 1.09	NR	NR
Alcohol use disorder	Azam, 2018	Pre-post surgery	Mix	2 years	cohort/case-control	6 (NR)	OR	REM	0.981	0.84 to 1.14	NR	NR
Anxiety symptoms	Loh,2021	Pre-post surgery	Mix	3-120 months	Cohort	26 (11255)	OR	FEM	0.58	0.51to 0.67	64.80%	None
Depressive symptoms	Loh,2021	Pre-post surgery	Mix	3-120 months	Cohort	30 (11255)	OR	REM	0.49	0.37 to 0.65	86%	None
Minimal depression	Alyahya, R. A.2022	Pre-post surgery	Mix	2-3years	cohort/case-control	3 (NR)	PR	REM	64.9	63.3 to 66.5	NR	None
Mild depression	Alyahya, R. A.2022	Pre-post surgery	Mix	1-3years	cohort/case-control	6 (NR)	PR	REM	12.7	11.8 to 13.7	NR	None
Moderate depression	Alyahya, R. A.2022	Pre-post surgery	Mix	1-3years	cohort/case-control	6(NR)	PR	REM	5.1	4.4 to 5.8	NR	None
Severe depression	Alyahya, R. A.2022	Pre-post surgery	Mix	1-3years	cohort/case-control	6 (98757)	PR	REM	1.9	1.5 to 2.4	NR	None
Depression symptoms	Alyahya, R. A.2022	Pre-post surgery	Mix	6-45.6 months	cohort/case-control	27 (98757)	PR	REM	15.3	15 to 15.5	NR	None
Depression symptoms	Woods, R.2023	Pre-post surgery	Mix	6 months	cohort/case-control	19 (NR)	SMD	REM	0.806	0.66 to 0.96	83.60%	Yes
Depression symptoms	Woods, R.2023	Pre-post surgery	Mix	12 months	cohort/case-control	35 (NR)	SMD	REM	0.804	0.68 to 0.93	96.90%	Yes
Depression symptoms	Woods, R.2023	Pre-post surgery	Mix	24 months	cohort/case-control	13 (NR)	SMD	REM	0.801	0.66 to 0.94	89.20%	Yes
Depression symptoms	Fu, R.2022	Pre-post surgery	Mix	6-60 months	cohort/RCT	11 (924)	OR	REM	0.29	0.14 to 0.57	94%	None
Suicide mortality	Lim, R. B. C.2018	Pre-post surgery	Mix	12-216 months	cohort/case-control	61 (142356)	SMD	REM	0.3	0.3 to 0.4	66.20%	0.28
Suicide	Castaneda, 2019	Non-surgery	Mix	8-10 years	cohort/case-control	3 (132314)	OR	FEM	4.15	3.20 to 5.38	15%	None
Self-harm	Castaneda, 2019	Pre-post surgery	Mix	8-10 years	cohort/case-control	3 (43,406)	OR	REM	1.9	1.23 to 2.95	99%	None
Eating disorders	Taba, J. V.2021	Pre-post surgery	Mix	1-7 years	cohort/case-control	8 (NR)	PR	REM	7.83	4.30 to 11.37	85.60%	Yes
Binge-eating disorder	Taba, J. V.2021	Pre-post surgery	Mix	1-7 years	cohort/case-control	6 (NR)	PR	REM	3.81	1.98 to 5.64	46.60%	Yes

MA, meta-analyses; CI, confidence interval; IGB, intragastric balloon; RCT, randomised clinical trial; SMD, standardised mean difference; REM, random-effects model; NR, not reported; OR, odds ratio; FEM, fixed-effect model; PR, prevalence ratio.

### General mental health

**Figure 3:**
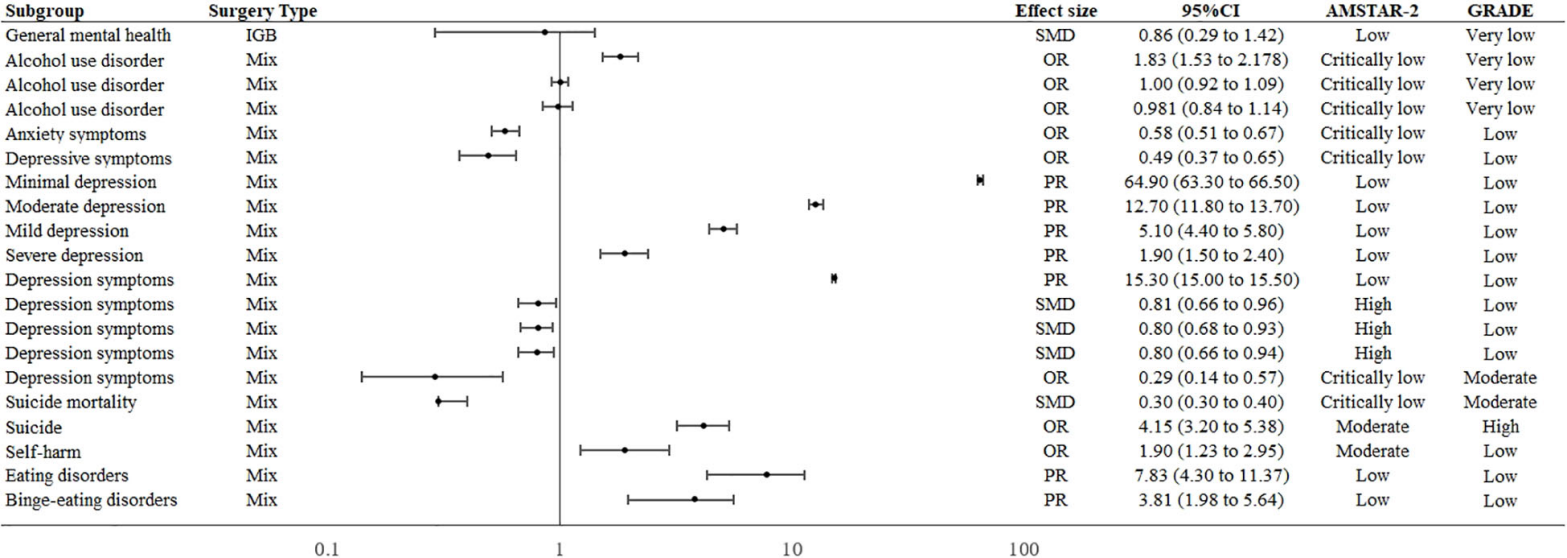
Forest plot of mental health outcomes associated with bariatric surgery.

For general mental health, a research report that a significant improvement could occur after an intragastric balloon (IGB) (OR = 0.86; 95% CI: 0.29 to 1.42) at six to 76 months of follow-up (35) (**Figure 3**).

### Depression and anxiety

The four studies included in this review all reported on the effect of bariatric surgery on reducing depression and anxiety symptoms (36–39). One study reported that bariatric surgery reducing anxiety (OR = 0.58; 95% CI: 0.51 to 0.67) (36). Two studies reported odds ratios for bariatric surgery to improve depression, OR = 0.29 (95% CI: 0.14 to 0.57) and OR = 0.49 (95% CI: 0.37 to 0.65) (36, 39). The prevalence of depression after bariatric surgery was as high as 15.3%, with severe, moderate, and mild depression accounting for 1.9% (95% CI: 1.5 to 2.4%), 5.1% (95% CI: 4.4 to5.8%), and 64.9% (95% CI: 63.3 to 66.5%) respectively (37). In addition, changes in depressive symptom scores were observed to have a large effect after bariatric surgery (SMD = 0.804, 95% CI: 0.73 to 0.88). Reductions in depressive symptom scores after bariatric surgery were comparable at follow-up: Variation in 6 months (SMD = 0.806; 95% CI: 0.66 to 0.96), 12 months (SMD = 0.804; 95% CI: 0.68 to 0.93), 24month(SMD = 0.801; 95% CI: 0.66 to 0.94) follow-up (38) (**Figure 3**).

### Self-harm and suicide

Bariatric surgery increases the risk of suicide (OR = 4.15; 95% CI: 3.20 to 5.38) and self-harm (OR = 1.90; 95% CI: 1.23 to 2.95), follow-up 8 to 10 years (40). (**Figure 3**) The suicide rate at 12-228 months after bariatric surgery is 0.3% (95% CI:0.3 to 0.4%) (41).

### Eating disorders

The overall postoperative prevalence of eating disorders is 7.83% (95% CI: 4.30 to 11.37). Considering the six studies that make up these, the prevalence of BED was 3.81% (17) (**Figure 3**).

### AUD

Bariatric surgery had no significant effect on AUD after 1 and 2 years of follow-up; However, the incidence of AUD was significantly higher after three years of follow-up (OR = 1.83; 95% CI: 1.53 to 2.178) (42) (**Figure 3**).

### AMSTAR-2 quality assessment

The methodological quality of the nine studies assessed was using the AMSTAR-2 tool. Most of the included reviews have weaknesses. Four studies (44.4%) were rated as critically low, three studies (33.3%) were rated as low, one study (11.1%) was rated as moderate, and one study (11.1%) was rated as high (**Table 4**).

**Table 4:** Methodological quality assessment of included meta-analyses according to the AMSTAR-2.

Author, year	1	2*	3	4*	5	6	7*	8	9*	10	11*	12	13*	14	15*	16	AMSTAR-2 grade
Alyahya, R. A. 2022	Y	PY	Y	Y	Y	N	PY	Y	N	N	Y	Y	Y	Y	Y	Y	Low
Azam, H. 2018	Y	PY	N	Y	N	Y	N	PY	Y	N	Y	Y	Y	N	N	Y	Critically low
Castaneda, D. 2019	Y	PY	N	PY	Y	Y	PY	Y	Y	N	Y	Y	Y	Y	Y	Y	Moderate
Fu, R. 2022	Y	N	Y	Y	Y	Y	PY	Y	Y	N	Y	N	N	Y	Y	N	Critically low
Gadd, N. 2020	Y	PY	Y	Y	Y	Y	Y	Y	Y	N	Y	Y	Y	PY	N	Y	Low
Lim, R. B. C. 2018	N	PY	N	PY	Y	Y	PY	PY	N	N	Y	N	N	Y	Y	Y	Critically low
Loh, H. H. 2021	Y	N	N	PY	Y	N	PY	Y	Y	N	Y	Y	Y	N	N	Y	Critically low
Taba, J. V. 2021	Y	Y	Y	Y	Y	Y	PY	Y	Y	N	Y	Y	Y	N	N	Y	Low
Woods, R. 2023	Y	Y	Y	Y	Y	Y	Y	Y	PY	N	Y	Y	Y	Y	Y	Y	High

* mean critical domains, Y, yes; PY, probably yes; N, no.

### GRADE classification

The GRADE system was used to evaluate the evidence quality of each postoperative mental health outcome. Only one outcome was rated as high quality (5.0%), two (10.0%) as moderate, thirteen (65.0%) as low, and four (20.0%) as very low (**Table 5**).

**Table 5:** GRADE quality assessment.

Outcome	Author, year	Type of bariatric surgery	Risk of bias	Inconsistency	Indirectness	Imprecision	Publication bias	Large effect	Dose-response gradient	All plausible confounding	Quality of evidence
General mental health	Gadd, N.2020	IGB	-1	-1	0	-1	NR	0	0	0	Very low
Alcohol use disorders	Azam, 2018	All types	-1	NR	0	-1	NR	0	0	0	Very low
Alcohol use disorders	Azam, 2018	All types	-1	NR	0	0	NR	0	0	0	Very low
Alcohol use disorders	Azam, 2018	All types	-1	NR	0	-1	NR	0	0	0	Very low
Anxiety symptoms	Loh, 2021	All types	0	-1	0	0	-1	0	0	0	Low
Depressive symptoms	Loh, 2021	All types	0	-1	0	0	-1	0	0	0	Low
Minimal depression	Alyahya, R. A.2022	All types	-1	-1	0	0	0	0	0	0	Low
Moderate depression	Alyahya, R. A.2022	All types	-1	-1	0	0	0	0	0	0	Low
Mild depression	Alyahya, R. A.2022	All types	-1	-1	0	0	0	0	0	0	Low
Severe depression	Alyahya, R. A.2022	All types	-1	-1	0	0	0	0	0	0	Low
Depression symptoms	Alyahya, R. A.2022	All types	-1	-1	0	0	0	0	0	0	Low
Depression symptoms	Woods, R.2023	All types	-1	-1	0	0	-1	1	0	0	Low
Depression symptoms	Woods, R.2023	All types	-1	-1	0	0	-1	1	0	0	Low
Depression symptoms	Woods, R.2023	All types	-1	-1	0	0	-1	1	0	0	Low
Depression symptoms	Fu, R.2022	All types	-1	-1	0	0	0	1	0	0	Moderate
Suicide mortality	Lim, R. B. C.2018	All types	0	-1	0	0	0	0	0	0	Moderate
Suicide	Castaneda, 2019	All types	-1	0	0	0	0	1	0	0	High
Self-harm	Castaneda, 2019	All types	-1	-1		0	0	0	0	0	Low
Eating disorders	Taba, J. V.2021	All types	-1	0	0	0	-1	0	0	0	Low
Binge-eating disorder	Taba, J. V.2021	All types	-1	0	0	0	-1	0	0	0	Low

NR mean not reported.

## Discussion

This umbrella review summarised the highest-level evidence on bariatric surgery for multiple mental health outcomes. We included nine meta-analyses/systemic reviews assessing 20 different mental health outcomes. This study found that bariatric surgery was beneficial to improving overall mental health, reducing depression and anxiety symptoms in patients with obesity, and eating disorders (e.g., BED) also improved after surgery. In addition, bariatric surgery can increase the risk of suicide, self-harm, and increased AUD after surgery. Although the mental state has improved after the operation, the mechanism behind this evidence is still unclear. It may not be bariatric surgery itself, but the life changes caused by weight loss (caused by bariatric surgery). The same is true of harmful consequences.

Bariatric surgery can significantly improve the symptoms of depression and anxiety and reduce the incidence; this has been proved in many meta-analyses (35, 36, 38, 39).

As expected, The degree of change in depressive symptoms is related to the shift in BMI after the operation (43). Although bariatric surgery can improve depressive symptoms in the short term, long-term follow-up studies show that the Hospital anxiety and depression scale is equivalent to before surgery in four years and nine years after bariatric surgery (44, 45). There may be several paths in its mechanism. (a) First, body image satisfaction, self-worth, interpersonal relationships (5), and an increase in physical activity can be improved after the operation (46, 47). Secondly, after surgery, changes in digestion or intestinal absorption may change biochemical signals in the brain; for example, changes in intestinal microflora, intestinal peptides (including ghrelin, glucagon-like peptide 1, peptide YY and cholecystokinin) are related to depression and anxiety, and bile acids can have a central impact on the emotional and behavioural responses caused by cocaine (48). Third, patients whose depression is likely to improve on their own are more likely to undergo surgery. Notably, studies have reported an 8% to 74% reduction in the prevalence of postoperative depression (5). However, the prevalence of postoperative depression is still as high as 15.3%, of which mild depression accounts for 64.9% (37); that may be related to weight regain following bariatric surgery, eating disorders, and impaired quality of life.

There was a significant improvement in BED (49, 50) after bariatric surgery, especially in the first 12 months after surgery, and larger effect sizes were found. One study included seven studies that reported the prevalence of eating disorders as 7.83% (17). Even if the postoperative prevalence is less than 10%, the disease can significantly affect prognosis and BMI reduction. Among them, the prevalence of BED is 3.81% (17). After bariatric surgery, avoiding high-fat/high-sugar foods may be a learned response to postprandial discomfort or dumping syndrome (51), this in turn helps to control obesity in this population (52). With longer postoperative follow-ups, conflicting outcomes emerged. Smith et al. (53) reported that BED increased from 2.1% to 4% after seven years, and loss of control eating increased from 24.6% to 26.4%. Conversely, Kalarchian et al. (54) report that the prevalence of eating disorders drops to 0% within seven years. The reason may be that preoperative comprehensive nutrition and psychological behaviour evaluation, education, and support decreased with time (55).

Notably, bariatric surgery is associated with an increased risk of suicide, self-harm, and AUD (40, 42). The prevalence of suicide after bariatric surgery was 0.3% (41). Peterhansel et al. (19) who reviewed suicide after bariatric surgery, reported that suicide rates were estimated at 4.1 per 10,000 people per year, with above-average suicide rates in bariatric surgery patients. Lack of improvement in quality of life after surgery, persistent or recurrent sexual dysfunction and interpersonal problems, and limited physical activity can all lead to an increased risk of suicide (20). The degree of BMI reduction does not seem to explain the change in mental distress after bariatric surgery (56). Compared with other patients undergoing surgery, the patients who have committed suicide or attempted suicide have similar or more significant BMI reduction (57). People with pre-existing mental illnesses such as depression and eating disorders are more likely to commit suicide after surgery due to the underlying psychiatric effects of people with obesity (58). The surgery itself increases potential postoperative problems, such as difficulty controlling pain leading to substance abuse, complications requiring further treatment, dissatisfaction with BMI reduction, excess skin, and scarring (59–61). Neuroendocrine changes and nutritional deficiency caused by postoperative malabsorption and/or irregular eating behaviour may also increase the risk of suicide (62). Because of the potential symptom change after the operation (cross-addiction hypothesis: from overeating to excessive drinking), it may be related to depressive symptoms and suicide attempts, which leads to an increase in the risk of AUD (63, 64). Bariatric surgery is associated with a significant increase in moderate to high-risk AUD (42). One study reported an 8% incidence of postoperative alcohol use/abuse; patients were more likely to drink heavily after surgery than before surgery, 19% had significant alcohol consumption before bariatric surgery, and 23% had apparent alcoholism after bariatric surgery (65). Of the three studies that reported rates of alcohol abuse (23, 66, 67), two studies reported an increase in alcohol problems after RYGB (23, 67), another study reported that no alcohol problems were found in LAGB (66). Increased alcohol consumption after RYGB may be due to physiological changes after gut bypass that are absent after LAGB (including increased release of glucagon-like peptide 1 and peptide YY after meals and decreased circulating acyl-ghrelin) (68, 69); these changes contribute to changes in neural activity (70). Intestinal flora may affect mood and behaviour, and exposure to abused drugs, such as alcohol and opioids, will induce intestinal microbial ecological imbalance (48).

In addition, we found no evidence to support a link between personality disorders and PTSD and changes in BMI reduction outcomes. Depression and anxiety are common among candidates for bariatric surgery. About 40% of patients with obesity have underlying psychiatric disorders, and early identification and optimisation of these disorders are important because they can affect surgical outcomes (71). In the future, we still need to pay attention to the mental health of patients with obesity and assess their mental health status before and after surgery.

### Limitations

Our umbrella review was limited to evidence included in the review search window, creating limitations regarding study timeliness. Those with more severe depressive symptoms may not be eligible for bariatric surgery or cannot participate in studies and/or stop participating in longitudinal studies (72, 73). It is unclear whether the increased risk of mental health problems after bariatric surgery is due to the surgery or the factors that force them to seek surgical treatment. The relationship between bariatric interventions and psychiatric outcomes is likely to be confounded by psychosocial features, limiting the value of inferences drawn from observational studies. Due to a lack of relevant data, we did not separately analyse the effects of different bariatric surgeries on mental health, and different BMI reduction methods were associated with other mental health outcomes. We should investigate the relationship between different BMI reduction methods and mental health-related outcomes in the future. In addition, the methodological quality of the meta-analysis we included is not high to the evidence of the outcome and stratified results. There is a need to track bariatric surgery’s mental health-related outcomes in real-time and summarise the latest evidence.

## Conclusion

We found that bariatric surgery effectively improved mental health and most mental health-related outcomes, such as anxiety symptoms, depression symptoms, eating disorders, and BED. However, these should be cautiously approached after bariatric surgery due to the increased risk of unfavourable mental suicide, self-harm, and AUD.

## Author contributions

SL: Conceptualization, Data curation, Formal Analysis, Methodology, Project administration, Software, Writing – original draft, Writing – review & editing. SD: Data curation, Investigation, Methodology, Software, Supervision, Writing – original draft. FZ: Data curation, Investigation, Methodology, Software, Validation, Writing – original draft. DZ: Conceptualization, Data curation, Formal Analysis, Investigation, Methodology, Project administration, Writing – review & editing. CW: Formal Analysis, Methodology, Project administration, Supervision, Validation, Writing – review & editing. ZD: Conceptualization, Data curation, Formal Analysis, Project administration, Resources, Supervision, Writing – review & editing.

## References

[1] LeBlanc EL Patnode CD Webber EM Redmond N Rushkin M O’Connor EA. Behavioral and pharmacotherapy weight loss interventions to prevent obesity-related morbidity and mortality in adults: an updated systematic review for the U.S. preventive services task force. Rockville (MD): Agency for Healthcare Research and Quality (US). (2018). 30354042

[2] Curry SJ Krist AH Owens DK Barry MJ Caughey AB Davidson KW. Behavioral weight loss interventions to prevent obesity-related morbidity and mortality in adults: US preventive services task force recommendation statement. Jama (2018) 320(11):1163–71. doi: 10.1001/jama.2018.13022 30326502

[3] Yaylali G Tekekoglu S Akin F. Sexual dysfunction in obese and overweight women. Int J Impot Res (2010) 22(4):220–6. doi: 10.1038/ijir.2010.7 20485360

[4] Wimmelmann CL Lund R Flensborg-Madsen T Christensen U Osler M Mortensen EL. Associations of personality with body mass index and obesity in a large late midlife community sample. Obes Facts (2018) 11(2):129–43. doi: 10.1159/000487888 PMC5981669 29631276

[5] Dawes AJ Maggard-Gibbons M Maher AR Booth MJ Miake-Lye I Beroes JM. Mental health conditions among patients seeking and undergoing bariatric surgery a meta-analysis. JAMA (2016) 315(2):150–63. doi: 10.1001/jama.2015.18118 26757464

[6] Cox S Brode C. Predictors of binge eating among bariatric surgery candidates: disinhibition as a mediator of the relationship between depressive symptoms and binge eating. Obes Surg (2018) 28(7):1990–6. doi: 10.1007/s11695-018-3129-8 PMC6019622 29411242

[7] Minhas M Murphy CM Balodis IM Samokhvalov AV MacKillop J. Food addiction in a large community sample of Canadian adults: prevalence and relationship with obesity, body composition, quality of life and impulsivity. Addict (Abingdon England) (2021) 116(10):2870–9. doi: 10.1111/add.15446 33843091

[8] Cazzo E Pareja JC Chaim EA Geloneze B Barreto MR Magro DO. GLP-1 and GLP-2 levels are correlated with satiety regulation after roux-en-Y gastric bypass: results of an exploratory prospective study. Obes Surg (2017) 27(3):703–8. doi: 10.1007/s11695-016-2345-3 27565666

[9] Al-Alsheikh AS Alabdulkader S Johnson B Goldstone AP Miras AD. Effect of obesity surgery on taste. Nutrients (2022) 14(4):866. doi: 10.3390/nu14040866 35215515 PMC8878262

[10] de Almeida Godoy CM de Araújo Quadros Cunha B Furtado MC de Godoy EP de Souza LBR Oliveira AG. Relationship of food intolerance 2 years after roux-en-Y gastric bypass surgery for obesity with masticatory efficiency and protein consumption. Obes Surg (2020) 30(8):3093–8. doi: 10.1007/s11695-020-04669-z 32415633

[11] Nielsen MS Schmidt JB le Roux CW Sjödin A. Effects of roux-en-Y gastric bypass and sleeve gastrectomy on food preferences and potential mechanisms involved. Curr Obes Rep (2019) 8(3):292–300. doi: 10.1007/s13679-019-00354-0 31222526

[12] Arterburn DE Olsen MK Smith VA Livingston EH Van Scoyoc L Yancy WS Jr. Association between bariatric surgery and long-term survival. Jama (2015) 313(1):62–70. doi: 10.1001/jama.2014.16968 25562267

[13] Campos GM Khoraki J Browning MG Pessoa BM Mazzini GS Wolfe L. Changes in utilization of bariatric surgery in the United States from 1993 to 2016. Ann Surg (2020) 271(2):201–9. doi: 10.1097/SLA.0000000000003554 31425292

[14] Chao GF Bonham AJ Ross R Stricklen A Ghaferi AA. Patient-reported comorbidity assessment after bariatric surgery: A potential tool to improve longitudinal follow-up. Ann Surg (2022) 276(6):e792–e7. doi: 10.1097/SLA.0000000000004841 33914479

[15] Kubik JF Gill RS Laffin M Karmali S. The impact of bariatric surgery on psychological health. J Obes (2013), 837989. doi: 10.1155/2013/837989 23606952 PMC3625597

[16] Ribeiro G Giapietro HB Belarmino LB Salgado-Junior W. Depression, anxiety, and binge eating before and after bariatric surgery: problems that remain. Arq Bras Cir Dig (2018) 31(1):e1356. doi: 10.1590/0102-672020180001e1356 29947690 PMC6050001

[17] Taba JV Suzuki MO Nascimento FSD Iuamoto LR Hsing WT Pipek LZ. The development of feeding and eating disorders after bariatric surgery: A systematic review and meta-analysis. Nutrients (2021) 13(7):2396. doi: 10.3390/nu13072396 34371904 PMC8308796

[18] Kalarchian MA Marcus MD. Psychosocial concerns following bariatric surgery: current status. Curr Obes Rep (2019) 8(1):1–9. doi: 10.1007/s13679-019-0325-3 30659459

[19] Peterhänsel C Petroff D Klinitzke G Kersting A Wagner B. Risk of completed suicide after bariatric surgery: a systematic review. Obes Rev (2013) 14(5):369–82. doi: 10.1111/obr.12014 23297762

[20] Mitchell JE Crosby R de Zwaan M Engel S Roerig J Steffen K. Possible risk factors for increased suicide following bariatric surgery. Obes (Silver Spring Md) (2013) 21(4):665–72. doi: 10.1002/oby.20066 PMC4372842 23404774

[21] Konttinen H Sjöholm K Jacobson P Svensson PA Carlsson LMS Peltonen M. Prediction of suicide and nonfatal self-harm after bariatric surgery: A risk score based on sociodemographic factors, lifestyle behavior, and mental health: A nonrandomized controlled trial. Ann Surg (2021) 274(2):339–45. doi: 10.1097/SLA.0000000000003742 PMC7283001 31850987

[22] Gordon KH King WC White GE Belle SH Courcoulas AP Ebel FE. A longitudinal examination of suicide-related thoughts and behaviors among bariatric surgery patients. Surg Obes related Dis (2019) 15(2):269–78. doi: 10.1016/j.soard.2018.12.00 PMC6481310 31010651

[23] King WC Chen JY Mitchell JE Kalarchian MA Steffen KJ Engel SG. Prevalence of alcohol use disorders before and after bariatric surgery. Jama (2012) 307(23):2516–25. doi: 10.1001/jama.2012.6147 PMC3682834 22710289

[24] Castaneda D Popov VB Wander P Thompson CC. Risk of suicide and self-harm is increased after bariatric surgery-a systematic review and meta-analysis. Obes Surg (2019) 29(1):322–33. doi: 10.1007/s11695-018-3493-4 30343409

[25] Orellana ER Covasa M Hajnal A. Neuro-hormonal mechanisms underlying changes in reward related behaviors following weight loss surgery: Potential pharmacological targets. Biochem Pharmacol (2019) 164:106–14. doi: 10.1016/j.bcp.2019.04.004 30954487

[26] Aromataris E Fernandez R Godfrey CM Holly C Khalil H Tungpunkom P. Summarizing systematic reviews: methodological development, conduct and reporting of an umbrella review approach. Int J Evidence-Based healthcare (2015) 13(3):132–40. doi: 10.1097/XEB.0000000000000055 26360830

[27] Ioannidis JP. Integration of evidence from multiple meta-analyses: a primer on umbrella reviews, treatment networks and multiple treatments meta-analyses. CMAJ (2009) 181(8):488–93. doi: 10.1503/cmaj.081086 PMC2761440 19654195

[28] Balk EM Chung M Chen ML Trikalinos TA Kong Win Chang L. Assessing the accuracy of google translate to allow data extraction from trials published in non-english languages. Rockville (MD): Agency for Healthcare Research and Quality (US) (2013) Jan. Report No.: 12(13)-EHC145-EF. 23427350

[29] Page MJ McKenzie JE Bossuyt PM Boutron I Hoffmann TC Mulrow CD. The PRISMA 2020 statement: an updated guideline for reporting systematic reviews. BMJ (Clinical Res ed) (2021) 372:n71. doi: 10.1136/bmj.n71 PMC8005924 33782057

[30] Shea BJ Reeves BC Wells G Thuku M Hamel C Moran J. AMSTAR 2: a critical appraisal tool for systematic reviews that include randomised or non-randomised studies of healthcare interventions, or both. BMJ (Clinical Res ed) (2017) 358:j4008. doi: 10.1136/bmj.j4008 PMC5833365 28935701

[31] Guyatt GH Oxman AD Kunz R Woodcock J Brozek J Helfand M. GRADE guidelines: 7. Rating the quality of evidence–inconsistency. J Clin Epidemiol (2011) 64(12):1294–302. doi: 10.1016/j.jclinepi.2011.03.017 21803546

[32] Guyatt G Oxman AD Akl EA Kunz R Vist G Brozek J. GRADE guidelines: 1. Introduction-GRADE evidence profiles and summary of findings tables. J Clin Epidemiol (2011) 64(4):383–94. doi: 10.1016/j.jclinepi.2010.04.026 21195583

[33] Hennessy EA Johnson BT. Examining overlap of included studies in meta-reviews: Guidance for using the corrected covered area index. Res synthesis Methods (2020) 11(1):134–45. doi: 10.1002/jrsm.1390 PMC8555740 31823513

[34] Pieper D Antoine S-L Mathes T Neugebauer EAM Eikermann M. Systematic review finds overlapping reviews were not mentioned in every other overview. Clin Epidemiol (2014) 67(4):368–75. doi: 10.1016/j.jclinepi.2013.11.007 24581293

[35] Gadd N McIntosh A Fear-Keen B Hoult J Maimone IR Marshall S. Do endoscopic bariatric procedures improve postprocedural quality of life and mental health? A systematic review and meta-analysis. Obes Surg (2020) 30(10):4091–100. doi: 10.1007/s11695-020-04860-2 32761319

[36] Loh HH Francis B Lim LL Lim QH Yee A Loh HS. Improvement in mood symptoms after post-bariatric surgery among people with obesity: A systematic review and meta-analysis. Diabetes/metabolism Res Rev (2021) 37(8):e3458. doi: 10.1002/dmrr.3458 PMC9285936 33891377

[37] Alyahya RA Alnujaidi MA. Prevalence and outcomes of depression after bariatric surgery: A systematic review and meta-analysis. Cureus (2022) 14(6):e25651. doi: 10.7759/cureus.25651 35784972 PMC9249077

[38] Woods R Moga AM Ribeiro PAB Stojanovic J Lavoie KL Bacon SL. Evolution of depressive symptoms from before to 24 months after bariatric surgery: A systematic review and meta-analysis. Obes Rev (2023) 24(5):e13557. doi: 10.1111/obr.13557 36823768

[39] Fu R Zhang Y Yu K Mao D Su H. Bariatric surgery alleviates depression in obese patients: A systematic review and meta-analysis. Obes Res Clin Pract (2022) 16(1):10–6. doi: 10.1016/j.orcp.2021.11.002 34802982

[40] Castaneda D Popov VB Wander P Thompson CC. Risk of suicide and self-harm is increased after bariatric surgery—a systematic review and meta-analysis. Obes Surg (2019) 29(1):322–33. doi: 10.1007/s11695-018-3493-4 30343409

[41] Lim RBC Zhang MWB Ho RCM. Prevalence of all-cause mortality and suicide among bariatric surgery cohorts: A meta-analysis. Int J Environ Res Public Health (2018) 15(7):1519. doi: 10.3390/ijerph15071519 30021983 PMC6069254

[42] Azam H Shahrestani S Phan K. Alcohol use disorders before and after bariatric surgery: A systematic review and meta-analysis. Ann Trans Med (2018) 6(8):148. doi: 10.21037/atm.2018.03.16 PMC5952017 29862237

[43] Mitchell JE King WC Chen JY Devlin MJ Flum D Garcia L. Course of depressive symptoms and treatment in the longitudinal assessment of bariatric surgery (LABS-2) study. Obes (Silver Spring Md) (2014) 22(8):1799–806. doi: 10.1002/oby.20738 PMC4115026 24634371

[44] Herpertz S Müller A Burgmer R Crosby RD de Zwaan M Legenbauer T. Health-related quality of life and psychological functioning 9 years after restrictive surgical treatment for obesity. Surg Obes related Dis (2015) 11(6):1361–70. doi: 10.1016/j.soard.2015.04.008 26164111

[45] Burgmer R Legenbauer T Müller A de Zwaan M Fischer C Herpertz S. Psychological outcome 4 years after restrictive bariatric surgery. Obes Surg (2014) 24(10):1670–8. doi: 10.1007/s11695-014-1226-x 24682804

[46] King WC Chen JY Belle SH Courcoulas AP Dakin GF Elder KA. Change in pain and physical function following bariatric surgery for severe obesity. Jama (2016) 315(13):1362–71. doi: 10.1001/jama.2016.3010 PMC4856477 27046364

[47] Rosenberger PH Henderson KE White MA Masheb RM Grilo CM. Physical activity in gastric bypass patients: associations with weight loss and psychosocial functioning at 12-month follow-up. Obes Surg (2011) 21(10):1564–9. doi: 10.1007/s11695-010-0283-z PMC3652016 20890771

[48] Brown RM Guerrero-Hreins E Brown WA le Roux CW Sumithran P. Potential gut-brain mechanisms behind adverse mental health outcomes of bariatric surgery. Nat Rev Endocrinol (2021) 17(9):549–59. doi: 10.1038/s41574-021-00520-2 34262156

[49] White MA Kalarchian MA Masheb RM Marcus MD Grilo CM. Loss of control over eating predicts outcomes in bariatric surgery patients: a prospective, 24-month follow-up study. J Clin Psychiatry (2010) 71(2):175–84. doi: 10.4088/JCP.08m04328blu PMC2831110 19852902

[50] Kalarchian MA King WC Devlin MJ Marcus MD Garcia L Chen JY. Psychiatric disorders and weight change in a prospective study of bariatric surgery patients: A 3-year follow-up. Psychosom Med (2016) 78(3):373–81. doi: 10.1097/PSY.0000000000000277 PMC5041300 26569540

[51] Istfan NW Lipartia M Anderson WA Hess DT Apovian CM. Approach to the patient: management of the post-bariatric surgery patient with weight regain. J Clin Endocrinol Metab (2021) 106(1):251–63. doi: 10.1210/clinem/dgaa702 PMC7765654 33119080

[52] Praxedes DRS Silva-Júnior AE Macena ML Oliveira AD Cardoso KS Nunes LO. Prevalence of food addiction determined by the Yale Food Addiction Scale and associated factors: A systematic review with meta-analysis. Eur eating Disord Rev (2022) 30(2):85–95. doi: 10.1002/erv.2878 34953001

[53] Smith KE Orcutt M Steffen KJ Crosby RD Cao L Garcia L. Loss of control eating and binge eating in the 7 years following bariatric surgery. Obes Surg (2019) 29(6):1773–80. doi: 10.1007/s11695-019-03791-x PMC6948918 30820886

[54] Kalarchian MA King WC Devlin MJ Hinerman A Marcus MD Yanovski SZ. Mental disorders and weight change in a prospective study of bariatric surgery patients: 7 years of follow-up. Surg Obes related Dis (2019) 15(5):739–48. doi: 10.1016/j.soard.2019.01.008 PMC7045720 30826244

[55] Ostovan MA Zibaeenezhad MJ Keshmiri H Shekarforoush S. The impact of education on weight loss in overweight and obese adults. Int Cardiovasc Res J (2013) 7(3):79–82. 24757627 PMC3987437

[56] Dagsland V Andenæs R Karlsen TI. Generic health-related quality of life may not be associated with weight loss 4 years after bariatric surgery: a cross-sectional study. Obes Surg (2018) 28(10):3142–50. doi: 10.1007/s11695-018-3332-7 29968186

[57] Neovius M Bruze G Jacobson P Sjöholm K Johansson K Granath F. Risk of suicide and non-fatal self-harm after bariatric surgery: results from two matched cohort studies. Lancet Diabetes Endocrinol (2018) 6(3):197–207. doi: 10.1016/S2213-8587(17)30437-0 29329975 PMC5932484

[58] Roizblatt A Roizblatt D Soto-Aguilar BF. Suicide risk after bariatric surgery. Rev Med Chile (2016) 144(9):1171–6. doi: 10.1007/s11920-019-1069-1 28060979

[59] Fazel S Wolf A Palm C Lichtenstein P. Violent crime, suicide, and premature mortality in patients with schizophrenia and related disorders: a 38-year total population study in Sweden. Lancet Psychiatry (2014) 1(1):44–54. doi: 10.1016/S2215-0366(14)70223-8 25110636 PMC4124855

[60] Tindle HA Omalu B Courcoulas A Marcus M Hammers J Kuller LH. Risk of suicide after long-term follow-up from bariatric surgery. Am J Med (2010) 123(11):1036–42. doi: 10.1016/j.amjmed.2010.06.016 PMC4296730 20843498

[61] Klockhoff H Näslund I Jones AW. Faster absorption of ethanol and higher peak concentration in women after gastric bypass surgery. Br J Clin Pharmacol (2002) 54(6):587–91. doi: 10.1046/j.1365-2125.2002.01698.x PMC1874483 12492605

[62] Lu CW Chang YK Lee YH Kuo CS Chang HH Huang CT. Increased risk for major depressive disorder in severely obese patients after bariatric surgery - a 12-year nationwide cohort study. Ann Med (2018) 50(7):605–12. doi: 10.1080/07853890.2018.1511917 30101619

[63] Boenisch S Bramesfeld A Mergl R Havers I Althaus D Lehfeld H. The role of alcohol use disorder and alcohol consumption in suicide attempts–a secondary analysis of 1921 suicide attempts. Eur Psychiatry (2010) 25(7):414–20. doi: 10.1016/j.eurpsy.2009.11.007 20627467

[64] Blum K Bailey J Gonzalez AM Oscar-Berman M Liu Y Giordano J. Neuro-genetics of reward deficiency syndrome (RDS) as the root cause of “Addiction transfer”: A new phenomenon common after bariatric surgery. J Genet syndromes Gene Ther (2011) 2012(1):S2–001. doi: 10.4172/2157-7412.S2-001 PMC3593106 23483116

[65] Nasser K Verhoeff K Mocanu V. New persistent opioid use after bariatric surgery: a systematic review and pooled proportion meta-analysis. Surg Endosc (2023) 37(1):703–714. doi: 10.1007/s00464-022-09291-x 35534738

[66] Hayden MJ Murphy KD Brown WA O’Brien PE. Axis I disorders in adjustable gastric band patients: the relationship between psychopathology and weight loss. Obes Surg (2014) 24(9):1469–75. doi: 10.1007/s11695-014-1207-0 24570091

[67] Svensson PA Anveden Å Romeo S Peltonen M Ahlin S Burza MA. Alcohol consumption and alcohol problems after bariatric surgery in the Swedish obese subjects study. Obes (Silver Spring Md) (2013) 21(12):2444–51. doi: 10.1002/oby.20397 23520203

[68] Thanos PK Subrize M Delis F Cooney RN Culnan D Sun M. Gastric bypass increases ethanol and water consumption in diet-induced obese rats. Obes Surg (2012) 22(12):1884–92. doi: 10.1007/s11695-012-0749-2 PMC3615544 22976430

[69] Polston JE Pritchett CE Tomasko JM Rogers AM Leggio L Thanos PK. Roux-en-Y gastric bypass increases intravenous ethanol self-administration in dietary obese rats. PloS One (2013) 8(12):e83741. doi: 10.1371/journal.pone.0083741 24391816 PMC3877092

[70] Goldstone AP Miras AD Scholtz S Jackson S Neff KJ Pénicaud L. Link between increased satiety gut hormones and reduced food reward after gastric bypass surgery for obesity. J Clin Endocrinol Metab (2016) 101(2):599–609. doi: 10.1210/jc.2015-2665 26580235 PMC4880130

[71] Yen YC Huang CK Tai CM. Psychiatric aspects of bariatric surgery. Curr Opin Psychiatry (2014) 27(5):374–9. doi: 10.1097/YCO.0000000000000085 PMC4162326 25036421

[72] Sockalingam S Cassin S Crawford SA Pitzul K Khan A Hawa R. Psychiatric predictors of surgery non-completion following suitability assessment for bariatric surgery. Obes Surg (2013) 23(2):205–11. doi: 10.1007/s11695-012-0762-5 22961685

[73] Mechanick JI Apovian C Brethauer S Timothy Garvey W Joffe AM Kim J. Clinical practice guidelines for the perioperative nutrition, metabolic, and nonsurgical support of patients undergoing bariatric procedures - 2019 update: cosponsored by american association of clinical endocrinologists/american college of endocrinology, the obesity society, american society for metabolic and bariatric surgery, obesity medicine association, and american society of anesthesiologists. Obes (Silver Spring Md) (2020) 28(4):O1–o58. doi: 10.1016/j.soard.2019.10.025 32202076

