# Supplementary material for: Bariatric surgery and mental health outcomes: an umbrella review
Source: Front Endocrinol (Lausanne). 2023 Nov 2;14:1283621. doi: 10.3389/fendo.2023.1283621 (PMC10653334; doi:10.3389/fendo.2023.1283621)
Supplement: Supplementary file 1 [file DataSheet_1.pdf]

Search queries

Based on input from our panel, we defined mental health conditions as depression, anxiety, posttraumatic stress disorder PTSD, personality disorders, substance abuse disorders, suicidality, or suicidal ideation, and eating disorders—primarily binge eating disorder. Eating behaviors not classified as disorders (eg, grazing and cognitive restraint) and cigarette smoking were outside our scope.

PubMed：118

#6	Search: **((((((((Anxiety[MeSH Terms]) OR (Anxiety[Title/Abstract])) OR (Angst[Title/Abstract])) OR (Social Anxiety[Title/Abstract])) OR (Anxieties, Social[Title/Abstract])) OR (Anxiety, Social[Title/Abstract])) OR (Social Anxieties[Title/Abstract])) OR (Hypervigilance[Title/Abstract])) OR (Nervousness[Title/Abstract])) OR (Anxiousness[Title/Abstract])**	283,415	06:50:59
#5	Search: **((((((Depression[MeSH Terms]) OR (Depressive Symptoms[Title/Abstract])) OR (Depression[Title/Abstract])) OR (Depressive Symptom[Title/Abstract])) OR (Symptom, Depressive[Title/Abstract])) OR (Emotional Depression[Title/Abstract])) OR (Depression, Emotional[Title/Abstract])**	497,521	06:49:11
#4	Search: **((((((((((((((((Mental Disorders[MeSH Terms]) OR (Mental Disorders[Title/Abstract])) OR (Mental Disorder[Title/Abstract])) OR (Psychiatric Illness[Title/Abstract])) OR (Psychiatric Illnesses[Title/Abstract])) OR (Psychiatric Diseases[Title/Abstract])) OR (Psychiatric Disease[Title/Abstract])) OR (Mental Illness[Title/Abstract])) OR (Illness, Mental[Title/Abstract])) OR (Mental Illnesses[Title/Abstract])) OR (Psychiatric Disorders[Title/Abstract])) OR (Psychiatric Disorder[Title/Abstract])) OR (Behavior Disorders[Title/Abstract])) OR (Diagnosis, Psychiatric[Title/Abstract])) OR (Psychiatric Diagnosis[Title/Abstract])) OR (Mental Disorders, Severe[Title/Abstract])) OR (Mental Disorder, Severe[Title/Abstract])) OR (Severe Mental Disorder[Title/Abstract])) OR (Severe Mental Disorders[Title/Abstract])**	1,463,241	06:47:28
#2	Search: **((((Mental health[MeSH Terms]) OR (Mental health[Title/Abstract])) OR (Mental Hygiene[Title/Abstract])) OR (Health, Mental[Title/Abstract])) OR (Hygiene, Mental[Title/Abstract])**	233,620	06:44:36
#1	Search: **((((((((((((bariatric surgery[MeSH Terms]) OR (bariatric surgery[Title/Abstract])) OR (metabolic surgery[Title/Abstract])) OR (laparoscopic sleeve gastrectomy[Title/Abstract])) OR (LSG[Title/Abstract])) OR (SG[Title/Abstract])) OR (laparoscopic Roux-en-Y gastric bypass[Title/Abstract])) OR (LRYGB[Title/Abstract])) OR (RYGB[Title/Abstract])) OR (laparoscopic adjustable gastric banding[Title/Abstract])) OR (one-anastomosis gastric bypass[Title/Abstract])) OR (OAGB[Title/Abstract])) OR (gastric bypass[Title/Abstract]))**	54,901	06:43:01
#0	Search: **Clipboard**	118	07:32:28

	[illegible]		
#9	Search: **((((Suicidal Ideation[MeSH Terms]) OR (Suicidal Ideation[Title/Abstract])) OR (Ideation, Suicidal[Title/Abstract])) OR (Ideations, Suicidal[Title/Abstract])) OR (Suicidal Ideations[Title/Abstract])**	19,665	06:59:14
#8	Search: **(((((((((((((((Personality Disorders[MeSH Terms]) OR (Personality Disorders[Title/Abstract])) OR (Personality Disorder[Title/Abstract])) OR (Avoidant Personality Disorder[Title/Abstract])) OR (Avoidant Personality Disorders[Title/Abstract])) OR (Personality Disorder, Avoidant[Title/Abstract])) OR (Personality Disorders, Avoidant[Title/Abstract])) OR (Inadequate Personality[Title/Abstract])) OR (Personality, Inadequate[Title/Abstract])) OR (Narcissistic Personality Disorder[Title/Abstract])) OR (Personality Disorder, Narcissistic[Title/Abstract])) OR (As If Personality[Title/Abstract])) OR (Personality, As If[Title/Abstract])) OR (Impulse-Ridden Personality[Title/Abstract])) OR (Impulse Ridden Personality[Title/Abstract])) OR (Personality, Impulse-Ridden[Title/Abstract])**	120,387	06:58:16
#7	Search: **((((((((((((((((((((((((((Stress Disorders, Post-Traumatic[MeSH Terms]) OR (Stress Disorder, Post-Traumatic[Title/Abstract])) OR (Post-Traumatic Stress Disorder[Title/Abstract])) OR (Stress Disorder, Post-Traumatic[Title/Abstract])) OR (Neuroses, Post-Traumatic[Title/Abstract])) OR (Neuroses, Post Traumatic[Title/Abstract])) OR (Post-Traumatic Neuroses[Title/Abstract])) OR (PTSD[Title/Abstract])) OR (Neuroses, Posttraumatic[Title/Abstract])) OR (Posttraumatic Neuroses[Title/Abstract])) OR (Post-Traumatic Stress Disorders[Title/Abstract])) OR (Post Traumatic Stress Disorders[Title/Abstract])) OR (Posttraumatic Stress Disorders[Title/Abstract])) OR (Posttraumatic Stress Disorder[Title/Abstract])) OR (Stress Disorder, Posttraumatic[Title/Abstract])) OR (Stress Disorders, Posttraumatic[Title/Abstract])) OR (Post Traumatic Stress Disorder[Title/Abstract])) OR (Stress Disorder, Post Traumatic[Title/Abstract])) OR (Delayed Onset Post-Traumatic Stress Disorder[Title/Abstract])) OR (Delayed Onset Post Traumatic Stress Disorder[Title/Abstract])) OR (Chronic Post-Traumatic Stress Disorder[Title/Abstract])) OR (Chronic Post Traumatic Stress Disorder[Title/Abstract])) OR (Moral Injury[Title/Abstract])) OR (Injury, Moral[Title/Abstract])) OR (Moral Injuries[Title/Abstract])) OR (Acute Post-Traumatic Stress Disorder[Title/Abstract])) OR (Acute Post Traumatic Stress Disorder[Title/Abstract])**	56,446	06:55:28

#15	Search: **#1 AND #14 AND #13**	118	07:30:00
#14	Search: **#2 OR #4 OR #5 OR #6 OR #7 OR #8 OR #9 OR #10 OR #11**	2,136,573	07:19:52
#13	Search: **(((((((((Meta-Analysis as Topic[MeSH Terms])) OR (Meta-Analysis[Publication Type])) OR (Systematic Review[Publication Type])) OR (Systematic Reviews as Topic[MeSH Terms])) OR (meta-analysis[Title/Abstract])) OR (meta-analyses[Title/Abstract])) OR (data pooling[Title/Abstract])) OR (clinical trial overview[Title/Abstract])) OR (clinical trial overviews[Title/Abstract])**	408,464	07:17:33
#11	Search: **(((((((((((((((((((((((((((((((((((((((Substance-Related Disorders[MeSH Terms]) OR (Substance-Related Disorders[Title/Abstract])) OR (Substance Related Disorder[Title/Abstract])) OR (Disorder, Substance Related[Title/Abstract])) OR (Disorders, Substance Related[Title/Abstract])) OR (Related Disorder, Substance[Title/Abstract])) OR (Related Disorders, Substance[Title/Abstract])) OR (Drug Use Disorders[Title/Abstract])) OR (Drug Use Disorder[Title/Abstract])) OR (Disorder, Drug Use[Title/Abstract])) OR (Organic Mental Disorders, Substance-Induced[Title/Abstract])) OR (Organic Mental Disorders, Substance Induced[Title/Abstract])) OR (Substance Abuse[Title/Abstract])) OR (Abuse, Substance[Title/Abstract])) OR (Substance Abuses[Title/Abstract])) OR (Substance Dependence[Title/Abstract])) OR (Dependence, Substance[Title/Abstract])) OR (Substance Addiction[Title/Abstract])) OR (Addiction, Substance[Title/Abstract])) OR (Chemical Dependence[Title/Abstract])) OR (Chemical Dependences[Title/Abstract])) OR (Dependence, Chemical[Title/Abstract])) OR (Dependences, Chemical[Title/Abstract])) OR (Drug Dependence[Title/Abstract])) OR (Dependence, Drug[Title/Abstract])) OR (Drug Addiction[Title/Abstract])) OR (Addiction, Drug[Title/Abstract])) OR (Prescription Drug Abuse[Title/Abstract])) OR (Abuse, Prescription Drug[Title/Abstract])) OR (Drug Abuse, Prescription[Title/Abstract])) OR (Substance Use[Title/Abstract])) OR (Substance Uses[Title/Abstract])) OR (Use, Substance[Title/Abstract])) OR (Drug Abuse[Title/Abstract])) OR (Abuse, Drug[Title/Abstract])) OR (Drug Habituation[Title/Abstract])) OR (Habituation, Drug[Title/Abstract])) OR (Substance Use Disorders[Title/Abstract])) OR (Disorder, Substance Use[Title/Abstract])) OR (Substance Use Disorder[Title/Abstract])**	432,642	07:05:19
#10	Search: **(((((Binge-Eating Disorder[MeSH Terms]) OR (Binge-Eating Disorder[Title/Abstract])) OR (Binge Eating Disorder[Title/Abstract])) OR (Binge-Eating Disorders[Title/Abstract])) OR (Disorder, Binge-Eating[Title/Abstract])) OR (Disorders, Binge-Eating[Title/Abstract])**	4,220	07:00:43

WOS：188

#1 TS=Bariatric surgery OR metabolic surgery OR laparoscopic sleeve gastrectomy OR LSG OR SG OR laparoscopic Roux-en-Y gastric bypass OR LRYGB OR RYGB OR laparoscopic adjustable gastric banding OR one-anastomosis gastric bypass OR OAGB OR gastric bypass

#2 TS=Mental health OR Mental Hygiene

#3 TS=Mental Disorders OR Mental Disorder OR Psychiatric Illness OR Psychiatric Illnesses OR Psychiatric Diseases OR Psychiatric Disease OR Mental Illness OR Mental Illnesses OR Psychiatric Disorders OR Psychiatric Disorder OR Behavior Disorders OR Psychiatric Diagnosis OR Mental Disorders OR Severe Mental Disorders

#4 TS=Depression OR Depressive Symptoms OR Depressive Symptom OR Symptom, Depressive OR Emotional Depression OR Depression, Emotional OR Mental Health OR Health, Mental OR Mental Hygiene OR Hygiene, Mental

#5 TS=Anxiety OR Angst OR Social Anxiety OR Anxieties, Social OR Anxiety OR Social Anxieties OR Hypervigilance OR Nervousness OR Anxiousness

#6 TS=posttraumatic stress disorder OR PTSD OR Stress Disorders, Post-Traumatic

#7 TS=Personality Disorders OR Personality Disorder OR Avoidant Personality Disorder OR Avoidant Personality Disorders OR Personality Disorder OR Avoidant OR Personality Disorders OR Avoidant, Inadequate Personality OR Personality OR Inadequate, Narcissistic Personality Disorder

#8 TS=Suicidal Ideation OR Ideation, Suicidal OR Ideations, Suicidal OR Suicidal Ideations

#9 TS=Binge-Eating Disorder OR Binge Eating Disorder OR Binge-Eating Disorders OR Disorder, Binge-Eating OR Disorders, Binge-Eating OR eating disorder* OR eating behavio* OR maladaptive eating

#10 TS=Substance-Related Disorders OR Substance Related Disorder

TS=Meta-Analysis as Topic OR Meta-Analysis OR Systematic Review OR Systematic Reviews as Topic OR meta-analysis OR meta-analyses OR data pooling OR clinical trial overview OR clinical trial overviews)

#11 TS=meta analysis OR meta analysis OR meta-analysis OR metaanalysis OR meta-analysis as topic OR metaanalyses OR systematic review OR systematic review OR meta-analysis OR metaanalysis

#12 #2 OR #3 OR #4 OR #5 OR #6 OR #7 OR #8 OR #9 OR #10

#13 #1 AND #12 AND #11---------------------------- Search：188

Embase：792

Bariatric surgery

'metabolic surgery' OR 'laparoscopic sleeve gastrectomy' OR 'lsg' OR 'sg' OR 'laparoscopic roux-en-y gastric bypass' OR 'lrygb' OR 'rygb' OR 'laparoscopic adjustable gastric banding' OR 'one-anastomosis gastric bypass' OR 'oagb' OR 'gastric bypass'

Mental health

'condition, mental' OR 'health, mental' OR 'mental care' OR 'mental condition' OR 'mental factor' OR 'mental help' OR 'mental service' OR 'mental state' OR 'mental status' OR 'mental status schedule' OR 'psychic health'

Mental disease

'abnormal mental state' OR 'disease, mental' OR 'diseased mental state' OR 'disorder, mental' OR 'disordered mental state' OR 'disturbed mental state' OR 'illness, mental' OR 'insanity' OR 'mental abnormality' OR 'mental change' OR 'mental confusion' OR 'mental defect' OR 'mental disorder' OR 'mental disorders' OR 'mental disorders diagnosed in childhood' OR 'mental disturbance' OR 'mental illness' OR 'mental insufficiency' OR 'mental symptom' OR 'mentally ill' OR 'neurodevelopmental disorder' OR 'neurodevelopmental disorders' OR 'neuropsychiatric disease' OR 'neuropsychiatric diseases' OR 'neuropsychiatric disorder' OR 'neuropsychiatric disorders' OR 'psychiatric disease' OR 'psychiatric disorder' OR 'psychiatric illness' OR 'psychiatric symptom' OR 'psychic disease' OR 'psychic disorder' OR 'psychic disturbance' OR 'psychologic disorder' OR 'psychologic disturbance' OR 'psychological disorder' OR 'psychological disturbance' OR 'psychopathology'

Depression

'central depression' OR 'clinical depression' OR 'depressive disease' OR 'depressive disorder' OR 'depressive episode' OR 'depressive illness' OR 'depressive personality disorder' OR 'depressive state' OR 'depressive symptom' OR 'depressive syndrome' OR 'mental depression' OR 'parental depression'

Posttraumatic stress disorder

'post-traumatic stress' OR 'post-traumatic stress disorder' OR 'post-traumatic stress disorders' OR 'posttraumatic neurosis' OR 'posttraumatic psychic syndrome' OR 'posttraumatic psychosis' OR 'posttraumatic stress' OR 'posttraumatic syndrome' OR 'psychosis, posttraumatic' OR 'PTSD' OR 'PTSD (posttraumatic stress disorder)' OR 'stress disorders, post-traumatic' OR 'stress disorders, traumatic' OR 'stress, posttraumatic' OR 'trauma and stressor related disorders' OR 'traumatic stress' OR 'traumatic stress disorder' OR 'traumatic stress disorders'

Personality disorder

'personality change' OR 'personality disorders' OR 'personality disturbance'

Suicidal ideation
'ideation, suicidal' OR 'suicidal thought' OR 'suicidal thoughts' OR 'suicide ideation' OR 'suicide thought' OR 'suicide thoughts'
Binge eating disorder
'binge eating syndrome' OR 'binge overeating' OR 'binge-eating disorder' OR 'overeating, binge'
D**rug dependence**
'addict' OR 'addiction, drug' OR 'dependence, drug' OR 'drug addict' OR 'drug addiction' OR 'drug dependence model' OR 'drug dependency' OR 'drug facilitation' OR 'drug habituation' OR 'drug physical dependence' OR 'physical dependence' OR 'substance addiction' OR 'substance dependence' OR 'substance dependency' OR 'substance use disorder' OR 'substance use disorders' OR 'substance-related disorder' OR 'substance-related disorders' OR 'toxicomania' OR 'toxicomanias' OR 'toxicomanie'

Anxiety

Meta
'meta analysis (topic)' OR 'meta analysis' OR 'analysis, meta' OR 'meta-analysis' OR 'metaanalysis' OR 'meta-analysis as topic' OR 'metaanalyses' OR 'systematic review (topic)' OR 'systematic review' OR 'review, systematic' OR 'analysis, meta' OR 'meta-analysis' OR 'metaanalysis'

History Save | Delete | Print view | Export | Email Combine > using (•) And ( ) Or ^ Collapse

#14	#2 AND #12 AND #13	792
#13	#3 OR #4 OR #5 OR #6 OR #7 OR #8 OR #9 OR #10 OR #11	3,021,992
#12	'meta analysis (topic)'/exp OR 'meta analysis (topic)' OR 'meta analysis' OR 'meta-analysis as topic' OR 'metaanalyses' OR 'systematic review (topic)' OR 'systematic review' OR 'review, systematic' OR 'analysis, meta' OR 'meta-analysis' OR 'metaanalysis'	679,152
#11	'anxiety'/exp	282,817
#10	'drug dependence'/exp OR 'addict' OR 'addiction, drug' OR 'dependence, drug' OR 'drug addict' OR 'drug addiction' OR 'drug dependence model' OR 'drug dependency' OR 'drug facilitation' OR 'drug habituation' OR 'drug physical dependence' OR 'physical dependence' OR 'substance addiction' OR 'substance dependence' OR 'substance dependency' OR 'substance use disorder' OR 'substance use disorders' OR 'substance-related disorder' OR 'substance-related disorders' OR 'toxicomania' OR 'toxicomanias' OR 'toxicomanie'	322,998
#9	'binge eating disorder'/exp OR 'binge eating syndrome' OR 'binge overeating' OR 'binge-eating disorder' OR 'overeating, binge'	9,586
#8	'suicidal ideation'/exp OR 'ideation, suicidal' OR 'suicidal thought' OR 'suicidal thoughts' OR 'suicide ideation' OR 'suicide thought' OR 'suicide thoughts'	32,397
#7	'personality disorder'/exp OR 'personality change' OR 'personality disorders' OR 'personality disturbance'	77,145
#6	'posttraumatic stress disorder'/exp OR 'post-traumatic stress' OR 'post-traumatic stress disorder' OR 'post-traumatic stress disorders' OR 'posttraumatic neurosis' OR 'posttraumatic psychic syndrome' OR 'posttraumatic psychosis' OR 'posttraumatic stress' OR 'posttraumatic syndrome' OR 'psychosis, posttraumatic' OR 'ptsd' OR 'ptsd (posttraumatic stress disorder)' OR 'stress disorders, post-traumatic' OR 'stress disorders, traumatic' OR 'stress, posttraumatic' OR 'trauma and stressor related disorders' OR 'traumatic stress' OR 'traumatic stress disorder' OR 'traumatic stress disorders'	90,624
#5	'depression'/exp OR 'central depression' OR 'clinical depression' OR 'depressive disease' OR 'depressive disorder' OR 'depressive episode' OR 'depressive illness' OR 'depressive personality disorder' OR 'depressive state' OR 'depressive symptom' OR 'depressive syndrome' OR 'mental depression' OR 'parental depression'	624,071
#4	'mental disease'/exp OR 'abnormal mental state' OR 'disease, mental' OR 'diseased mental state' OR 'disorder, mental' OR 'disordered mental state' OR 'disturbed mental state' OR 'illness, mental' OR 'insanity' OR 'mental abnormality' OR 'mental change' OR 'mental confusion' OR 'mental defect' OR 'mental disorder' OR 'mental disorders' OR 'mental disorders diagnosed in childhood' OR 'mental disturbance' OR 'mental illness' OR 'mental insufficiency' OR 'mental symptom' OR 'mentally ill' OR 'neurodevelopmental disorder' OR 'neurodevelopmental disorders' OR 'neuropsychiatric disease' OR 'neuropsychiatric diseases' OR 'neuropsychiatric disorder' OR 'neuropsychiatric disorders' OR 'psychiatric disease' OR 'psychiatric disorder' OR 'psychiatric illness' OR 'psychiatric symptom' OR 'psychic disease' OR 'psychic disorder' OR 'psychic disturbance' OR 'psychologic disorder' OR 'psychologic disturbance' OR 'psychological disorder' OR 'psychological disturbance' OR 'psychopathology'	2,774,574
#3	'mental health'/exp OR 'condition, mental' OR 'health, mental' OR 'mental care' OR 'mental condition' OR 'mental factor' OR 'mental help' OR 'mental service' OR 'mental state' OR 'mental status' OR 'mental status schedule' OR 'psychic health'	296,074
#2	'bariatric surgery'/exp OR 'metabolic surgery' OR 'laparoscopic sleeve gastrectomy' OR 'lsg' OR 'sg' OR 'laparoscopic roux-en-y gastric bypass' OR 'lrygb' OR 'rygb' OR 'laparoscopic adjustable gastric banding' OR 'one-anastomosis gastric bypass' OR 'oagb' OR 'gastric bypass'	164,374
#1	'bariatric surgery'/de	40,655

Cochrane library: 20

MeSH term - Bariatric Surgery

(Bariatric surgery):ti,ab,kw OR (metabolic surgery):ti,ab,kw OR (laparoscopic sleeve gastrectomy):ti,ab,kw OR (LSG):ti,ab,kw OR (SG):ti,ab,kw OR (laparoscopic Roux-en-Y gastric bypass):ti,ab,kw OR (LRYGB):ti,ab,kw OR (RYGB):ti,ab,kw OR (laparoscopic adjustable gastric banding):ti,ab,kw OR (one-anastomosis gastric bypass):ti,ab,kw OR (OAGB):ti,ab,kw OR (gastric bypass):ti,ab,kw

MeSH term - Mental Health

(Mental Health):ti,ab,kw OR (Hygiene):ti,ab,kw OR (Mental):ti,ab,kw OR (Mental):ti,ab,kw OR (Mental Hygiene):ti,ab,kw

MeSH term - Mental Disorders

(Mental Disorders):ti,ab,kw OR (Diagnosis, Psychiatric):ti,ab,kw OR (Psychiatric Diagnosis):ti,ab,kw OR (Behavior Disorders):ti,ab,kw OR (Mental Disorders, Severe):ti,ab,kw OR (Severe Mental Disorder):ti,ab,kw OR (Severe Mental Disorders):ti,ab,kw OR (Mental Disorder, Severe):ti,ab,kw OR (Psychiatric Illness):ti,ab,kw OR (Psychiatric Illnesses):ti,ab,kw OR (Psychiatric Disease):ti,ab,kw OR (Psychiatric Diseases):ti,ab,kw OR (Illness, Mental):ti,ab,kw OR (Mental Illness):ti,ab,kw OR (Psychiatric Disorder):ti,ab,kw OR (Mental Illnesses):ti,ab,kw OR (Mental Disorder):ti,ab,kw OR (Psychiatric Disorders):ti,ab,kw

MeSH term - Depression

(Depression):ti,ab,kw OR (Emotional Depression):ti,ab,kw OR (Symptom, Depressive):ti,ab,kw OR (Depressive Symptom):ti,ab,kw OR (Depressive Symptoms):ti,ab,kw OR (Depression, Emotional):ti,ab,kw

MeSH term - Anxiety

(Anxiety):ti,ab,kw OR (Anxieties, Social):ti,ab,kw OR (Anxiety, Social):ti,ab,kw OR (Social Anxiety):ti,ab,kw OR (Social Anxieties):ti,ab,kw OR (Hypervigilance):ti,ab,kw OR (Anxiousness):ti,ab,kw OR (Angst):ti,ab,kw OR (Nervousness):ti,ab,kw

MeSH term- posttraumatic stress disorder

(posttraumatic stress disorder):ti,ab,kw OR (Acute Post-Traumatic Stress Disorder):ti,ab,kw OR (Acute Post Traumatic Stress Disorder):ti,ab,kw OR (Chronic Post-Traumatic Stress Disorder):ti,ab,kw OR (Chronic Post Traumatic Stress Disorder):ti,ab,kw OR (Posttraumatic Stress Disorder):ti,ab,kw OR (Post

Traumatic Stress Disorders):ti,ab,kw OR (Stress Disorder, Posttraumatic):ti,ab,kw OR (Stress Disorders, Posttraumatic):ti,ab,kw OR (Stress Disorder, Post Traumatic):ti,ab,kw OR (Neuroses, Posttraumatic):ti,ab,kw OR (PTSD):ti,ab,kw OR (Neuroses, Post-Traumatic):ti,ab,kw OR (Post Traumatic Stress Disorder):ti,ab,kw OR (Post-Traumatic Stress Disorder):ti,ab,kw OR (Posttraumatic Stress Disorders):ti,ab,kw OR (Neuroses, Post Traumatic):ti,ab,kw OR (Post-Traumatic Neuroses):ti,ab,kw OR (Stress Disorder, Post-Traumatic):ti,ab,kw OR (Post-Traumatic Stress Disorders):ti,ab,kw OR (Posttraumatic Neuroses):ti,ab,kw OR (Delayed Onset Post Traumatic Stress Disorder):ti,ab,kw OR (Delayed Onset Post-Traumatic Stress Disorder):ti,ab,kw OR (Injury, Moral):ti,ab,kw OR (Moral Injuries):ti,ab,kw OR (Moral Injury):ti,ab,kw

MeSH term-Substance-Related Disorders

(Substance-Related Disorders):ti,ab,kw OR (Drug Dependence):ti,ab,kw OR (Addiction, Drug):ti,ab,kw OR (Dependence, Drug):ti,ab,kw OR (Drug Addiction):ti,ab,kw OR (Substance Addiction):ti,ab,kw OR (Dependences, Chemical):ti,ab,kw OR (Chemical Dependences):ti,ab,kw OR (Chemical Dependence):ti,ab,kw OR (Addiction, Substance):ti,ab,kw OR (Dependence, Substance):ti,ab,kw OR (Substance Dependence):ti,ab,kw OR (Dependence, Chemical):ti,ab,kw OR (Drug Abuse, Prescription):ti,ab,kw OR (Prescription Drug Abuse):ti,ab,kw OR (Abuse, Prescription Drug):ti,ab,kw OR (Related Disorder, Substance):ti,ab,kw OR (Related Disorders, Substance):ti,ab,kw OR (Disorders, Substance Related):ti,ab,kw OR (Disorder, Substance Related):ti,ab,kw OR (Substance Related Disorder):ti,ab,kw OR (Substance Use):ti,ab,kw OR (Use, Substance):ti,ab,kw OR (Substance Uses):ti,ab,kw OR (Disorder, Drug Use):ti,ab,kw OR (Drug Use Disorders):ti,ab,kw OR (Drug Use Disorder):ti,ab,kw OR (Abuse, Drug):ti,ab,kw OR (Drug Abuse):ti,ab,kw OR (Habituation, Drug):ti,ab,kw OR (Drug Habituation):ti,ab,kw OR (Organic Mental Disorders, Substance-Induced):ti,ab,kw OR (Organic Mental Disorders, Substance Induced):ti,ab,kw OR (Substance Abuse):ti,ab,kw OR (Substance Abuses):ti,ab,kw OR (Abuse, Substance):ti,ab,kw OR (Substance Use Disorders):ti,ab,kw OR (Substance Use Disorder):ti,ab,kw OR (Disorder, Substance Use):ti,ab,kw

MeSH term - Personality Disorders

(Personality Disorders):ti,ab,kw OR (Personality, Inadequate):ti,ab,kw OR (Inadequate Personality):ti,ab,kw OR (As If Personality):ti,ab,kw OR (Personality, As If):ti,ab,kw OR (Personality Disorder, Narcissistic):ti,ab,kw OR (Narcissistic Personality Disorder):ti,ab,kw OR (Personality Disorder):ti,ab,kw OR (Avoidant Personality Disorder):ti,ab,kw OR (Avoidant Personality Disorders):ti,ab,kw OR (Personality Disorder, Avoidant):ti,ab,kw OR (Personality Disorders, Avoidant):ti,ab,kw OR (Impulse Ridden Personality):ti,ab,kw OR (Personality, Impulse-Ridden):ti,ab,kw OR (Impulse-Ridden Personality):ti,ab,kw

MeSH term - Binge-Eating Disorder

(Binge-Eating Disorders):ti,ab,kw OR (Disorder, Binge-Eating):ti,ab,kw OR (Binge Eating Disorder):ti,ab,kw OR (Disorders, Binge-Eating):ti,ab,kw

MeSH term - Meta Analysis as Topic

(Meta Analysis as Topic):ti,ab,kw OR (Data Pooling):ti,ab,kw OR (Overviews, Clinical Trial):ti,ab,kw OR (Overview, Clinical Trial):ti,ab,kw OR (Clinical Trial Overviews):ti,ab,kw OR (Clinical Trial Overview):ti,ab,kw OR (Data Poolings):ti,ab,kw OR (Review, Systematic, systematic review, Reviews Systematic as Topic):ti,ab,kw OR (Systematic Review as Topic):ti,ab,kw

MeSH term - Suicidal Ideation

(Suicidal Ideation):ti,ab,kw OR (Ideations, Suicidal):ti,ab,kw OR (Ideation, Suicidal):ti,ab,kw OR (Suicidal Ideations):ti,ab,kw

View fewer lines | Print search history

#1	MeSH descriptor: [Bariatric Surgery] explode all trees	MeSH	1625
#2	(Bariatric surgery):ti,ab,kw OR (metabolic surgery):ti,ab,kw OR (laparoscopic sleeve gastrectomy):ti,ab,kw OR (LSG):ti,ab,kw OR (SG):ti,ab,kw OR (laparoscopic Roux-en-Y gastric bypass):ti,ab,kw OR (LRYGB):ti,ab,kw OR (RYGB):ti,ab,kw OR (laparoscopic adjustable gastric banding):ti,ab,kw OR (one-anastomosis gastric bypass):ti,ab,kw OR (OAGB):ti,ab,kw OR (gastric bypass):ti,ab,kw	Limits	9394
#3	#1 OR #2	Limits	9594
#4	MeSH descriptor: [Mental Health] explode all trees	MeSH	3532
#5	(Mental Health):ti,ab,kw OR (Hygiene):ti,ab,kw OR (Mental):ti,ab,kw OR (Mental):ti,ab,kw OR (Mental Hygiene):ti,ab,kw	Limits	86203
#6	#4 OR #5	Limits	86203
#7	MeSH descriptor: [Mental Disorders] explode all trees	MeSH	98049
#8	(Mental Disorders):ti,ab,kw OR (Diagnosis, Psychiatric):ti,ab,kw OR (Psychiatric Diagnosis):ti,ab,kw OR (Behavior Disorders):ti,ab,kw OR (Mental Disorders, Severe):ti,ab,kw OR (Severe Mental Disorder):ti,ab,kw OR (Severe Mental Disorders):ti,ab,kw OR (Mental Disorder, Severe):ti,ab,kw OR (Psychiatric Illness):ti,ab,kw OR (Psychiatric Illnesses):ti,ab,kw OR (Psychiatric Disease):ti,ab,kw OR (Psychiatric Diseases):ti,ab,kw OR (Illness, Mental):ti,ab,kw OR (Mental Illness):ti,ab,kw OR (Psychiatric Disorder):ti,ab,kw OR (Mental Illnesses):ti,ab,kw OR (Mental Disorder):ti,ab,kw OR (Psychiatric Disorders):ti,ab,kw	Limits	64311
#9	#7 OR #8	Limits	129035
#10	MeSH descriptor: [Depression] explode all trees	MeSH	18299
#11	(Depression):ti,ab,kw OR (Emotional Depression):ti,ab,kw OR (Symptom, Depressive):ti,ab,kw OR (Depressive Symptom):ti,ab,kw OR (Depressive Symptoms):ti,ab,kw OR (Depression, Emotional):ti,ab,kw	Limits	98314
#12	#10 OR #11	Limits	98314
#13	MeSH descriptor: [Stress Disorders, Post-Traumatic] explode all trees	MeSH	3620
#14	(posttraumatic stress disorder):ti,ab,kw OR (Acute Post-Traumatic Stress Disorder):ti,ab,kw OR (Acute Post Traumatic Stress Disorder):ti,ab,kw OR (Chronic Post-Traumatic Stress Disorder):ti,ab,kw OR (Chronic Post Traumatic Stress Disorder):ti,ab,kw OR (Posttraumatic Stress Disorder):ti,ab,kw OR (Post Traumatic Stress Disorders):ti,ab,kw OR (Stress Disorder, Posttraumatic):ti,ab,kw OR (Stress Disorders, Posttraumatic):ti,ab,kw OR (Stress Disorder, Post Traumatic):ti,ab,kw OR (Neuroses, Posttraumatic):ti,ab,kw OR (PTSD):ti,ab,kw OR (Neuroses, Post-Traumatic):ti,ab,kw OR (Post Traumatic Stress Disorder):ti,ab,kw OR (Post-Traumatic Stress Disorder):ti,ab,kw OR (Posttraumatic Stress Disorders):ti,ab,kw OR (Neuroses, Post Traumatic):ti,ab,kw OR (Post-Traumatic Neuroses):ti,ab,kw OR (Stress Disorder, Post-Traumatic):ti,ab,kw OR (Post-Traumatic Stress Disorders):ti,ab,kw OR (Posttraumatic Neuroses):ti,ab,kw OR (Delayed Onset Post Traumatic Stress Disorder):ti,ab,kw OR (Delayed Onset Post-Traumatic Stress Disorder):ti,ab,kw OR (Injury, Moral):ti,ab,kw OR (Moral Injuries):ti,ab,kw OR (Moral Injury):ti,ab,kw	Limits	8034

#	Search		Results
#15	#13 OR #14	Limits	8034
#16	MeSH descriptor: [Anxiety] explode all trees	MeSH	13016
#17	(Anxiety):ti,ab,kw OR (Anxieties, Social):ti,ab,kw OR (Anxiety, Social):ti,ab,kw OR (Social Anxiety):ti,ab,kw OR (Social Anxieties):ti,ab,kw OR (Hypervigilance):ti,ab,kw OR (Anxiousness):ti,ab,kw OR (Angst):ti,ab,kw OR (Nervousness):ti,ab,kw	Limits	69623
#18	#16 OR #17	Limits	69789
#19	MeSH descriptor: [Substance-Related Disorders] explode all trees	MeSH	19561
#20	(Substance-Related Disorders):ti,ab,kw OR (Drug Dependence):ti,ab,kw OR (Addiction, Drug):ti,ab,kw OR (Dependence, Drug):ti,ab,kw OR (Drug Addiction):ti,ab,kw OR (Substance Addiction):ti,ab,kw OR (Dependences, Chemical):ti,ab,kw OR (Chemical Dependences):ti,ab,kw OR (Chemical Dependence):ti,ab,kw OR (Addiction, Substance):ti,ab,kw OR (Dependence, Substance):ti,ab,kw OR (Substance Dependence):ti,ab,kw OR (Dependence, Chemical):ti,ab,kw OR (Drug Abuse, Prescription):ti,ab,kw OR (Prescription Drug Abuse):ti,ab,kw OR (Abuse, Prescription Drug):ti,ab,kw OR (Related Disorder, Substance):ti,ab,kw OR (Related Disorders, Substance):ti,ab,kw OR (Disorders, Substance Related):ti,ab,kw OR (Disorder, Substance Related):ti,ab,kw OR (Substance Related Disorder):ti,ab,kw OR (Substance Use):ti,ab,kw OR (Use, Substance):ti,ab,kw OR (Substance Uses):ti,ab,kw OR (Disorder, Drug Use):ti,ab,kw OR (Drug Use Disorders):ti,ab,kw OR (Drug Use Disorder):ti,ab,kw OR (Abuse, Drug):ti,ab,kw OR (Drug Abuse):ti,ab,kw OR (Habituation, Drug):ti,ab,kw OR (Drug Habituation):ti,ab,kw OR (Organic Mental Disorders, Substance-Induced):ti,ab,kw OR (Organic Mental Disorders, Substance Induced):ti,ab,kw OR (Substance Abuse):ti,ab,kw OR (Substance Abuses):ti,ab,kw OR (Abuse, Substance):ti,ab,kw OR (Substance Use Disorders):ti,ab,kw OR (Substance Use Disorder):ti,ab,kw OR (Disorder, Substance Use):ti,ab,kw	Limits	61742
#21	#19 OR #20	Limits	69083
#22	MeSH descriptor: [Meta-Analysis as Topic] explode all trees	MeSH	1447
#23	MeSH descriptor: [Systematic Reviews as Topic] explode all trees	MeSH	109
#24	(Meta Analysis as Topic):ti,ab,kw OR (Data Pooling):ti,ab,kw OR (Overviews, Clinical Trial):ti,ab,kw OR (Overview, Clinical Trial):ti,ab,kw OR (Clinical Trial Overviews):ti,ab,kw OR (Clinical Trial Overview):ti,ab,kw OR (Data Poolings):ti,ab,kw OR (Review, Systematic, systematic review, Reviews Systematic as Topic):ti,ab,kw OR (Systematic Review as Topic):ti,ab,kw	Limits	14055
#25	#22 OR #23 OR #24	Limits	14108
#26	MeSH descriptor: [Personality Disorders] explode all trees	MeSH	1728
#27	(Personality Disorders):ti,ab,kw OR (Personality, Inadequate):ti,ab,kw OR (Inadequate Personality):ti,ab,kw OR (As If Personality):ti,ab,kw OR (Personality, As If):ti,ab,kw OR (Personality Disorder, Narcissistic):ti,ab,kw OR (Narcissistic Personality Disorder):ti,ab,kw OR (Personality Disorder):ti,ab,kw OR (Avoidant Personality Disorder):ti,ab,kw OR (Avoidant Personality Disorders):ti,ab,kw OR (Personality Disorder, Avoidant):ti,ab,kw OR (Personality Disorders, Avoidant):ti,ab,kw OR (Impulse Ridden Personality):ti,ab,kw OR (Personality, Impulse-Ridden):ti,ab,kw OR (Impulse-Ridden Personality):ti,ab,kw	Limits	7779
#28	#26 OR #27	Limits	7806
#29	MeSH descriptor: [Suicidal Ideation] explode all trees	MeSH	1008
#30	(Suicidal Ideation):ti,ab,kw OR (Ideations, Suicidal):ti,ab,kw OR (Ideation, Suicidal):ti,ab,kw OR (Suicidal Ideations):ti,ab,kw	Limits	3101
#31	#29 OR #30	Limits	3101
#32	MeSH descriptor: [Binge-Eating Disorder] explode all trees	MeSH	412
#33	(Binge-Eating Disorder):ti,ab,kw OR (Binge-Eating Disorders):ti,ab,kw OR (Disorder, Binge-Eating):ti,ab,kw OR (Binge Eating Disorder):ti,ab,kw OR (Disorders, Binge-Eating):ti,ab,kw	Limits	1208
#34	#32 OR #33	Limits	1208
#35	#3 AND (#6 OR #9 OR #12 OR #15 OR #18 OR #21 OR #28 OR #31 OR #34) AND #25	Limits	20
#36	Type a search term or use the S or MeSH buttons to compose	S / MeSH / Limits	N/A

Clear all

Highlight orphan lines
